# Potential predictors affecting outcomes in a randomized controlled trial of support programs for parents of young adults with hazardous substance use

**DOI:** 10.1186/s13722-025-00582-x

**Published:** 2025-07-10

**Authors:** Ola Siljeholm, Anders Hammarberg

**Affiliations:** Centre for Psychiatry Research, Department of Clinical Neuroscience, Karolinska Institutet, & Stockholm Health Care Services, Region Stockholm, Stockholm, Sweden

**Keywords:** Community reinforcement and family training, Randomized controlled trial, Predictors of outcome, Concerned significant other, Hazardous substance use, Young adult, Parental support program, Cognitive behavioral treatment

## Abstract

**Background:**

There is a lack of research on which specific factors that predict positive outcomes in support programs for concerned significant others (CSOs) to individuals with substance use disorders (SUDs). The aim of the study was to investigate predictors of positive outcomes among young adults in the areas of: treatment seeking, decreased substance use, and improved parent-young adult relationships. Outcomes were assessed at 24-weeks following a randomized controlled trial (RCT) comparing Community Reinforcement and Family Training (CRAFT) and manualized counselling for parents of young adults (18-24 years) with hazardous substance use.

**Methods:**

A secondary analysis from an RCT including 113 parents (92% female) of young adults (87% male) recruited from two outpatient clinics for young adults in Stockholm, Sweden, subsequently nationwide. Clinical and sociodemographic predictors of treatment entry, substance use, and relationship happiness at 24 weeks follow-up were assessed using linear and logistic mixed-effects models.

**Results:**

Previous young adult treatment engagement and higher parental self-efficacy were significant predictors of treatment entry, explaining 16% of the variance. Baseline alcohol and substance use consistently predicted higher use over time across measures. Parental stress showed complex associations: higher stress predicted reduced substance use in some models but increased use in others. For the measure of relationship happiness, baseline scores were the strongest predictor.

**Conclusions:**

Taken together, the findings suggest promising targets for tailored support programs for parents of young adults with SUD, such as components designed to enhance parental self-efficacy.

**Trial registration:**

ISRCTN12212515 https://www.isrctn.com/ISRCTN12212515. Submitted November 7th, 2018, registered March 4th, 2019.

## Introduction

Substance use, including alcohol consumption, significantly contributes to the overall disease burden among young adults in developed nations, and Sweden is no exception [1–3]. The prevalence of alcohol and substance use (henceforth referred to as substance use) typically peaks during early adulthood, around the ages of 18 to 25 [3, 4]. For some individuals, this period is characterized by hazardous use, defined as substance use patterns that heightens the risk of adverse physiological, psychological, and social outcomes or the development of substance use disorders (SUDs) [4–7]. The latest Swedish figures suggest that 14% of young adults aged 17–29 used substances other than alcohol in the past year [3]. Furthermore, approximately 21.5% self-reported meeting the criteria for Alcohol Use Disorder (AUD) and 4% for SUD [3]. However, studies show that less than 10% of young adults with AUD/SUD ever seek treatment, often due to factors such as lack of motivation, resistance to treatment options, or fear of stigma and shame [8, 9]. Engaging in treatment for AUD or SUD yields significant benefits for both the individual undergoing treatment (e.g., decreased substance use, improved mental health) [10–12] and their concerned significant others (CSOs) [13, 14].

Being a CSO to an individual with SUD or hazardous substance use (identified patient, IP), such as a parent to a young adult, is associated with increased risks of psychological and physical health issues stemming from various factors such as financial strain, excessive worry, stigma, and feeling blamed by others for the IP’s substance use [15–19]. Community Reinforcement and Family Training (CRAFT) is a structured support program for CSOs rooted in the principles of cognitive behavioral therapy (CBT) and motivational interviewing (MI) [20]. Concerned significant others who participate in CRAFT learn strategies to modify their own behavior with three primary objectives: (1) enhancing their own well-being; (2) reducing IP substance use; and (3) encouraging IP treatment-seeking behavior. Previous trials have examined the efficacy of CRAFT in promoting treatment seeking among individuals with substance use disorders, reporting treatment entry rates ranging from 21% to 86%, alongside improvements in CSO mental health and reductions in substance/alcohol use [13, 21–28]. Other available support programs for CSOs include Al-Anon Facilitation Therapy (AFT), based on 12-step principles, which has been shown to improve CSOs’ quality of life and coping abilities [28, 29]. Additionally, programs like the 5-step method and Coping Skills Training (CST), which focus on coping strategies and CSO mental health, have demonstrated reductions in stress and depression and improved coping [30, 31].

Offering different support programs for CSOs of substance-using IPs has in several reviews shown positive outcomes for both the CSOs themselves (e.g., improved mental health, coping, and relational happiness) and the IP (e.g., treatment seeking and decreased substance use) [14, 32–34]. None of these reviews have, however, investigated predictors, such as sociodemographic and clinical characteristics of the parents or adolescents, of favorable outcomes. This lack is partly due to the challenges of compiling results from studies of diverse programs with different contents, participant characteristics, and research settings where the studies have been performed [14, 32, 34]. Most findings regarding predictors of positive outcomes in CSO programs come from secondary or exploratory analyses in CRAFT trials. A systematic review by Archer et al. (2020) covering all available CRAFT studies at the time found that one study by Meyers et al. [22] showed that parents of adult children with substance use problems were more likely than other CSO-IP relationships to achieve treatment entry [13]. In a German study, CSOs with the primary goal of getting their IP into treatment were more successful in achieving treatment entry than those focusing on their own well-being [24]. Further, a Danish study found that CSOs who informed their IPs about their CRAFT participation had significantly higher odds of achieving treatment entry [35].

The current study is a secondary analysis of data from a randomized controlled trial (RCT) comparing the efficacy of CRAFT with manualized counseling for parents of young adults (18–24 years) with hazardous substance use who refused treatment [36]. The RCT showed that 33% of CRAFT participants and 31% of counseling participants reported young adult treatment entry by the 24-week follow-up, with no significant difference between groups. Both groups reported reduced substance use and improved relationship satisfaction, with no difference between conditions. Post-trial interviews with participants in the CRAFT group revealed that parents felt that the program significantly improved their relationship with their young adult, which in turn played a critical role in reducing substance use and, in some cases, facilitating treatment entry [37].

Given the lack of research on which factors are involved in positive outcomes in CRAFT treatment - treatment entry, reduced substance use and improved relationship -, it is essential to investigate the predictors associated with these outcomes. Identifying predictive factors can help tailor support programs for parents, improving treatment outcomes and strengthening support systems for young adults with hazardous substance use.

### Objectives

The aim of the current study was to investigate predictors of outcomes for young adult treatment initiation, young adult alcohol- and substance use, and parent – young adult relationship happiness at 24-weeks follow-up in an RCT comparing Community Reinforcement and Family Training (CRAFT) and manualized counselling.

### Method

Secondary analysis of data from the RCT assessing the efficacy of CRAFT compared to manualized counseling for parents of treatment-refusing young adults (18–24 years) with hazardous substance use [36]. The primary outcome was treatment engagement among the young adults, with secondary outcomes including substance use and relationship happiness. The design included a baseline measure and follow-up at 6, 12, and 24 weeks.

This study was approved by the Swedish Ethical Review Authority (region of Stockholm), DNR 2018/1642-3, and was conducted in accordance with Good Clinical Practice and the Declaration of Helsinki.

#### Participants

The study period extended from October 2018 to November 2021, with participant recruitment taking place between October 2018 and May 2021 via social media and the two outpatient clinics in Stockholm where the interventions were delivered. Baseline screening and collection of clinical data was performed during visits to the study coordinator for the first 52 participants. Due to the COVID-19 pandemic, the procedure changed in March 2020. For the remaining 61 participants, baseline screening was conducted via video, using a tablet, smartphone, or computer. Follow-up interviews were also held via video, and self-reported data was mailed to the research coordinator in pre-paid envelopes. The shift to video also enabled recruitment to expand to a nationwide uptake in Sweden.

In the RCT, young adults with hazardous substance use were included to capture a broader spectrum of substance use patterns and avoid restricting the sample to those meeting SUD criteria. The main eligibility criteria for participation in the RCT were: (1) Identified hazardous substance use (including alcohol) in the young adult defined as a score of > 2 using the CRAFFT questionnaire (Car, Relax, Alone, Forget, Friends, Trouble) [38, 39]; (2) young adult refusing to enter treatment the last month; (3) parent and young adult in contact at least 40% of the days and (4) parent not showing problematic alcohol- or substance use. For further eligibility criteria, see [36]. After screening, 113 participants were randomized to either CRAFT (*n* = 58) or manualized counseling (*n* = 55).

### Interventions

Both interventions were conducted through individual sessions with the participant (and co-parent if relevant). The initial 52 participants received face-to-face sessions at two outpatient clinics for adolescents and young adults (Maria Youth) within the Stockholm Centre for Dependency Disorders and Stockholm Municipality, Stockholm, Sweden, while the remaining 61 participants had sessions via video. Therapists in both conditions had extensive experience working with young adults with hazardous substance use, as well as with their parents. The therapists were instructed to deliver weekly treatment sessions, ensuring all sessions were completed within a 14-week timeframe.

#### Community reinforcement and family training

The experimental intervention consisted of eight CRAFT sessions (45–60 min), with themes in accordance with the CRAFT manual [20] tailored for parents of young adults, through relevant case descriptions and substance-related facts. Key components included functional analysis, positive communication, positive reinforcement, parental well-being, and strategies to enhance young adult treatment entry.

#### Control condition

The manualized counseling program included five individual 45-minute sessions and one group psychoeducation session on drugs and brain development. Key components included validation of parental concerns, mapping the parents’ social support network, investigating the young adult’s social network, and encouraging family activities.

See [36] for a more thorough description of the two interventions.

### Measures

All data, including that regarding the young adults, were provided by the participating parents. Those who had not provided data at the 24-week follow-up were contacted by phone to report solely on the primary outcome treatment seeking in the young adult.

### Outcome measures

The following baseline- and outcome measures were included as variables in the predictor analyses together with delivery mode (face-to-face vs. video) and allocated condition (CRAFT vs. manualized counselling).

#### Baseline measures

Young adult age and parent age; young adult level of education, categorized as: (1) in secondary school/vocational school (reference variable); (2) in primary school/prematurely dropped out of primary school; (3) finished secondary school; and (4) prematurely dropped out of secondary school; young adult employment status, categorized as unemployed, studies or work; young adult habitation, categorized as: (1) living with the parent (reference variable); and (2) living every other week with parent/living alone/living with another person; parent-young adult contact frequency, measured as the number of days (0–30) on a normal month that the parent and young adult are in contact with each other; young adult in treatment 180–30 days before baseline, categorized as yes or no; parent relationship status categorized as: (1) married/partnership/cohabitant (reference variable); and (2) single/divorced/widow/widower; and young adult primary problem substance, categorized as alcohol, cannabis, or “other” (e.g., cocaine, opioid analgesics, amphetamines, benzodiazepines).

Variables to include in the analyses were selected to represent a range of individual, familial, and contextual factors that previous research have shown relevant for the outcomes of interest. Young adult characteristics, such as age, level of education, employment status, habitation, and prior treatment engagement, were included to capture developmental, socioeconomic, and experiential influences on substance use behavior and treatment engagement [4, 40]. Parental factors, including relationship status, and parent-young adult contact frequency, were chosen to reflect the potential role of family dynamics and support systems [22, 41].

#### Primary outcome measure

Participants reported whether young adults sought treatment for hazardous substance use, recorded as a binary variable (1 = did seek treatment; 0 = did not seek treatment). Treatment options included specialized addiction treatment, support groups, primary care, psychiatry, telephone support lines, internet-based, or other (specified in free-text). Given the wide variation in substance use severity in the expected sample of young adults, treatment entry was broadly defined to include a range of services - from specialized addiction care to lower-threshold options such as primary care or digital support - as these may serve as important first steps into the treatment system, even if not sufficient on their own.

#### Secondary outcome measures

Young adult frequency of alcohol use one month prior to each assessment was measured via a tailored TimeLine FollowBack (TLFB) [42] method, marking “A” for days with alcohol consumption, while quantity and patterns of binge drinking during the last 12 months was assessed using the Alcohol Use Disorders Identification Test-Consumption (AUDIT-C) [39, 43]. Substance use one month prior to each assessment point was measured via the tailored TLFB-version, marking “D” for drug use days. Substance use associated problems during the last 12 months was measured with the Drug Use Disorder Identification Test (DUDIT) [44, 45]. Participant mental health was evaluated using the Depression, Anxiety and Stress Scale (DASS-21) [46, 47]. Relationship happiness was assessed with the Relationship Happiness Scale (RHS) [25, 48]. Parental self-efficacy was measured by a 48-item questionnaire developed by Ulfsdotter et al. [49].

#### Outcomes in predictor analyses

The following outcomes from the 24-weeks follow-up were chosen as outcomes in the predictor analyses: young adult treatment entry, young adult use of alcohol (TLFB/AUDIT-C), young adult use of substances and severity of substance use (TLFB/DUDIT), and parent’s relationship happiness (RHS).

#### Statistical analyses

All statistical analyses and regression models were conducted by a statistician who was not part of the research team.

#### Power calculation

Sample size calculations were based on the primary outcome for the full RCT and have been presented in a previous publication [36].

### Statistical methods

Statistical analysis and mixed effects modelling were performed using R version 4.1.1. Multiple imputation by chained equations (MICE) was performed using the mice package version 3.16.0 in R.

#### Analytical plan

The primary outcome, treatment seeking, was analyzed using logistic regression at 24 weeks providing odds ratios (OR) for effect estimates. All other outcomes were analyzed using mixed linear regressions, which included all time points except baseline, while controlling for baseline value. A mixed model was used due to repeated measurements, and a random intercept for individuals was included. The variable days with alcohol use last month (TLFB) was highly skewed and was hence log-transformed. Associations are based on the log-transformed outcome.

To address missing data, multiple imputation (MICE) was applied to all independent variables with missing values. In the multiple imputation approach, missing values for predictors were imputed through chained equations, ensuring that all available data could be used in the analyses. The final model included all imputed data, allowing for estimation of the effect of the predictors more robustly, with the pooled results from five multiple datasets. Thus, all analyses were based on the full sample (*n* = 113).

For the predictor analyses, stepwise linear and logistic regression models were used. Selection was based on the Akaike Information Criterion (AIC), where a lower AIC indicates a better model fit. Non-significant variables were included if they improved the AIC. Alternative model selection strategies were considered, including F-test-based stepwise procedures and penalized regression methods, i.e., methods applying complexity penalties, such as Least Absolute Shrinkage and Selection Operator (LASSO). Stepwise regression guided by AIC was ultimately chosen due to its ability to balance model fit and parsimony, its suitability for use with multiply imputed data, and its clinical interpretability within the constraints of a modest sample size. Univariable models were estimated first, where each predictor was included in the model individually, followed by multivariable models, where multiple predictors were included simultaneously.

## Results

### Baseline characteristics

Baseline characteristics and observed outcome values for both participants and young adults are presented in Table 1. The participants were 92% female, had a mean age of 51.5 years, and reported levels of depression, anxiety and stress that were below clinical cutoffs according to population norms using DASS-21 [46]. Of the young adults, 87% were male and had a mean age of 20 years. Approximately 20% had engaged in treatment during the last six months, although not the last month before parent entering the study.

**Table 1:** Demographic and clinical characteristics at baseline

Parents	CRAFT *n* = 58	Counselling *n* = 55
Gender, female, n (%)	53 (91%)	51 (93%)
Age (years), mean (SD)	50.9 (5.3)	51.9 (6.6)
*Level of education*,* n (%)*		
University or college	38 (65%)	37 (65%)
Secondary school, vocational school	16 (28%)	14 (24.5%)
Elementary school	0 (0%)	1 (1.75%)
Other	2 (3.5%)	3 (5.3%)
*Source of income*,* n (%)*		
Employed or self-employed	51 (88%)	50 (88%)
Other (retirement pension, sickness/activity pay)	7 (12%)	5 (9%)
*Marital status*,* n (%)*		
Married/Cohabitant	43 (74%)	33 (60%)
Single/Divorced/Separated/Widowed	15 (26%)	22 (40%)

	Mean (SD)	Mean (SD)
DASS-21 Depression	7.3 (8.4)	7.3 (7.4)
DASS-21 Anxiety	2.8 (5.3)	3.7 (5.3)
DASS-21 Stress	10.9 (8.3)	12.3 (8.5)
Relationship Happiness Scale	35.55 (14.7)	34.7 (13.6)

Young adults	CRAFT	Counselling
Gender, male, n (%)	48 (83%)	50 (88%)
Age (years), mean (SD)	19.8 (1.86)	20.2 (2.1)
*Level of education*,* n (%)*		
In secondary school/vocational school	24 (21.2)	17 (15.0)
In primary school/dropped out of primary school	3 (2.7)	8 (7.1)
Finished secondary school	13 (11.5)	18 (15.9)
Prematurely dropped out of secondary school	18 (15.9)	12 (10.6)
*Employment*,* n (%)*		
Studies	21 (36%)	13 (24%)
Job	19 (33%)	21 (39%)
No employment	18 (31%)	20 (37%)
*Main problematic substance*,* n (%)*		
Cannabis	32 (55.2%)	33 (60%)
Alcohol	6 (10.3%)	5 (9%)
Other (Cocaine, opioid analgesics, amphetamines, benzodiazepines)	20 (34.5%)	17 (31%)
Engaged in treatment past six months*, n (%)	14 (24.1%)	9 (16.4%)

	Mean (SD)	Mean (SD)
Days with substance use past month	12.4 (12.8)	11.7 (10.8)
Days with alcohol use past month	6.45 (5.1)	9.2 (7.8)
DUDIT	17.4 (8.3)	17.7 (9.7)
AUDIT-C	5.6 (2.65)	5.8 (2.35)

*DASS-21* Depression Anxiety Stress Scale, *DUDIT* Drug Use Disorders Identification Test, *AUDIT-C* Alcohol Use Disorders Identification Test– Consumption

* Parents were asked if their young adult had engaged in treatment during the last six months, but the young adults were not allowed to have participated in treatment during the last month (see exclusion criteria).

### Predictors of treatment outcome

Results from the predictor analyses are presented in Tables 2, 3, 4 and 5.

### Young adult treatment entry

Regarding the primary outcome treatment seeking in the young adult, the univariable analysis (Table 2) identified that previous young adult treatment engagement 180 − 30 days before baseline increased the odds for treatment engagement within the study period (OR 3.96; CI 1.33–11.76; *p* = 0.014). In the multivariable model, previous treatment engagement (*p* = 0.006) and higher parental self-efficacy (*p* = 0.071) resulted in the best model of fit, explaining 16% of the variance for young adult treatment seeking.

**Table 2:** Univariable and multivariable predictors of young adult treatment seeking at 24 weeks post inclusion

Term	Univariable			Multivariable			
	OR	95%-CI	*P*-value	OR	95%-CI	*P*-value	R2
							0.16
Age young adult	1.16	(0.91–1.50)	0.232				
Age parent	0.94	(0.86–1.03)	0.206				
*Young adult level of education* Primary school/dropped out of primary school	1.42	(0.28–7.24)	0.671				
Finished secondary school	0.36	(0.09–1.53)	0.166				
Dropped out of secondary school	0.86	(0.24–3.10)	0.814				
*Employment young adult* Studies	0.78	(0.23–2.64)	0.690				
Work	0.62	(0.19–2.05)	0.428				
*Habitation young adult* Every other week with parent/alone/with other	0.80	(0.27–2.35)	0.677				
Contact frequency parent-young adult	1.01	(0.96–1.06)	0.714				
Young adult in treatment 180 − 30 days before baseline: yes	3.96	(1.33–11.76)	0.014	5.95	(1.71–20.71)	0.006	
Parent relationship status: Single/divorced/widow/widower	0.94	(0.34–2.59)	0.897				
Delivery mode: Face-to-face	2.29	(0.86–6.11)	0.097				
Condition: CRAFT	0.78	(0.29–2.11)	0.626				
Main problematic Substance: Alcohol	2.61	(0.45–15.26)	0.277				
Main problematic Substance: Other	2.20	(0.77–6.27)	0.139				
DASS 21 stress	1.01	(0.96–1.07)	0.668				
DASS 21 depression	1.02	(0.96–1.08)	0.578				
DASS 21 anxiety	1.01	(0.92–1.11)	0.866				
Parental self-efficacy	1.01	(1.00-1.02)	0.206	1.01	(1.00-1.03)	0.071	
Baseline days with substance use last month (TLFB)	1.00	(0.98–1.02)	0.965				
Baseline days with alcohol use last month (TLFB)	1.01	(0.99–1.04)	0.388				

Reference variables for categorical predictors: Young adult level of education: In secondary school/vocational school; Employment young adult: Unemployed; Habitation young adult: With the participant; Parent relationship status: Married/Partnership/Cohabitant; and Main problematic substance: Cannabis

### Young adult alcohol use

#### Days with alcohol use last month (TLFB)

The univariable analyses for days with young adult alcohol use revealed that the only predictor was baseline alcohol use, where a higher level of days with alcohol use last month at baseline predicted a higher level over time (*p* < 0.001) (Table 3a). The best fit for multivariable model included baseline value of days with alcohol use last month (*p* < 0.001), young adult age where higher age predicted lower alcohol use over time (*p* = 0.028) and young adult previous treatment engagement (*p* = 0.123), together explaining 18% of the variance (Table 3a).

#### Alcohol consumption according to AUDIT-C

The univariable analyses for the outcome of young adult alcohol consumption measured with AUDIT-C revealed several statistically significant predictors (Table 3b). A higher baseline value of AUDIT-C (*p* < 0.001), higher young adult age (*p* < 0.001), having finished secondary school (*p* < 0.001) or dropped out of secondary school (*p* = 0.020) (compared to being in secondary school/vocational school), living every other week with parent/alone/with other person (*p* = 0.048) (compared to living at home with parent) and having alcohol rated by parent as main problematic substance (*p* < 0.001) all individually predicted a higher AUDIT-C score over time (Table 3b). Being a student (*p* = 0.036) (compared to unemployed/unknown) and parent receiving support program face-to-face (*p* = 0.035) predicted a lower AUDIT-C score over time.

In the multivariable model, only baseline AUDIT-C score (*p* < 0.001) was included showing that a higher score at baseline predicted a higher score over time, explaining 54% of the variance (Table 3b).

**Table 3:** (**a**) Univariable and multivariable predictors of young adult alcohol use (TLFB) last month over 24 weeks post inclusion. (**b**) Univariable and multivariable predictors of young adult alcohol consumption (AUDIT-C) over 24 weeks post inclusion

a								b						
Term	Univariable			Multivariable				Univariable			Multivariable			
	Coef	95%-CI	*P*-value	Coef	95%-CI	*P*-value	R2*	Coef	95%-CI	*P*-value	Coef	95%-CI	*P*-value	R2*
							0.18							0.54
Days with alcohol use last month (TLFB)	0.04	(0.02–0.06)	< 0.001	0.05	(0.03–0.07)	< 0.001		-	-	-	-	-	-	
Baseline AUDIT-C	-	-	-	-	-	-		0.63	(0.53–0.73)	< 0.001	0.63	(0.53–0.73)	< 0.001	
Age young adult	-0.01	(-0.18-0.16)	0.869	-0.19	(-0.36–-0.02)	0.028		0.34	(0.15–0.53)	< 0.001				
Age parent	0.03	(-0.02-0.08)	0.227					0.03	(-0.04-0.09)	0.379				
*Young adult level of education* Primary school/dropped out of primary school	-0.55	(-1.70-0.60)	0.345					-0.20	(-1.50-1.10)	0.761				
Finished secondary school	0.75	(-0.06-1.56)	0.070					1.68	(0.78–2.59)	< 0.001				
Dropped out of secondary school	0.16	(-0.68-1.00)	0.706					1.10	(0.18–2.02)	0.020				
*Employment young adult* Studies	0.16	(-0.64-0.95)	0.698					-1.02	(-1.97–0.07)	0.036				
Work	0.45	(-0.31-1.21)	0.243					0.31	(-0.61-1.22)	0.507				
*Habitation young adult* Every other week with parent or alone or with other	0.17	(-0.60-0.94)	0.657					0.89	(0.01–1.78)	0.048				
Contact frequency parent-young adult	-0.00	(-0.04-0.03)	0.800					-0.04	(-0.09-0.00)	0.062				
Young adult in treatment 180 − 30 days before baseline: yes	-0.50	(-1.48-0.47)	0.303	-0.71	(-1.63-0.21)	0.123		0.24	(-0.76-1.24)	0.640				
Parent relationship status: Single/divorced/widow/widower	0.33	(-0.40-1.06)	0.378					0.64	(-0.17-1.46)	0.122				
Delivery mode: Face-to-face	-0.36	(-1.02-0.30)	0.282					-0.82	(-1.57–0.06)	0.035				
Condition: CRAFT	-0.44	(-1.12-0.24)	0.200					0.09	(-0.68-0.87)	0.815				
Main problematic Substance: Alcohol	0.63	(-0.48-1.74)	0.263					2.47	(1.22–3.72)	< 0.001				
Main problematic Substance: Other	-0.17	(-0.91-0.58)	0.656					0.86	(-0.02-1.75)	0.056				
DASS 21 stress	0.02	(-0.02-0.06)	0.329					0.02	(-0.03-0.07)	0.491				
DASS 21 depression	0.01	(-0.03-0.05)	0.737					0.02	(-0.04-0.07)	0.557				
DASS 21 anxiety	0.05	(-0.01-0.11)	0.094					0.00	(-0.07-0.07)	0.987				
Parental self-efficacy	0.00	(-0.01-0.00)	0.242					0.00	(-0.01-0.01)	0.717				

Reference variables for categorical predictors: Young adult level of education: In secondary school/vocational school; Employment young adult: Unemployed; Habitation young adult: With the participant; Parent relationship status: Married/Partnership/Cohabitant; and Main problematic substance: Cannabis.

* R2 square is pooled R-squares according to Snijders and Bosker (2012)

#### Days with substance use last month (TLFB)

For prediction of days with substance use in the univariable analyses, a higher baseline value predicted more frequent use over time (*p* < 0.001), while main problematic substance rated as alcohol (*p* = 0.006) or as other substance than cannabis (*p* = 0.033) predicted a lower number of days with substance use over time (Table 4a).

In the multivariable model, baseline value of number of days with substance use (*p* < 0.001) was included together with parental level of stress at baseline (*p* = 0.045) showing that higher levels of parental stress at baseline predicted lower young adult substance use in subsequent follow-ups, together explaining 31% of the variance of change over time (Table 4a).

**Table 4:** (**a**) Univariable and multivariable predictors of young adult substance use (TLFB) last month over 24 weeks post inclusion. (**b**) Univariable and multivariable predictors of young adult severity of substance use (DUDIT) over 24 weeks post inclusion

a								b						
Term	Univariable			Multivariable				Univariable			Multivariable			
	Coef	95%-CI	P-value	Coef	95%-CI	*P*-value	R2*	Coef	95%-CI	*P*-value	Coef	95%-CI	P-value	R2*
							0.31							0.38
Days with substance use last month (TLFB)	0.45	(0.32–0.57)	< 0.001	0.48	(0.33–0.62)	< 0.001		-	-	-	-	-	-	
Baseline DUDIT-score	-	-	-	-	-	-		0.55	(0.34–0.75)	< 0.001	0.47	(0.23–0.71)	0.002	
Age young adult	0.58	(-1.57-2.74)	0.596					0.76	(-0.10-1.62)	0.083	0.49	(-0.37-1.35)	0.246	
Age parent	-0.30	(-1.00-0.40)	0.397					-0.38	(-0.64–0.12)	0.004				
*Young adult level of education* Primary school/dropped out of primary school	-5.61	(-20.90-9.68)	0.470					3.77	(-2.11-9.65)	0.207				
Finished secondary school	0.28	(-10.51-11.08)	0.959					0.71	(-3.43-4.85)	0.735				
Dropped out of secondary school	5.56	(-5.53-16.65)	0.324					3.98	(-0.27-8.22)	0.066				
*Employment young adult* Studies	-0.64	(-11.33-10.05)	0.906					-3.44	(-7.35-0.47)	0.084				
Work	2.06	(-8.11-12.23)	0.690					-2.50	(-6.37-1.36)	0.203				
*Habitation young adult* Every other week with parent or alone or with other	-2.23	(-11.81-7.35)	0.646					4.29	(0.76–7.83)	0.018				
Contact frequency parent-young adult	0.02	(-0.41-0.45)	0.914					-0.16	(-0.32-0.00)	0.055				
Young adult in treatment 180 − 30 days before baseline: yes	-3.37	(-14.06-7.33)	0.535					5.52	(1.49–9.55)	0.008				
Parent relationship status: Single/divorced/widow/widower	-2.52	(-11.50-6.46)	0.581					-1.19	(-4.63-2.25)	0.496				
Delivery mode: Face-to-face	-4.87	(-13.25-3.50)	0.253					-0.57	(-3.86-2.72)	0.732				
Condition: CRAFT	-0.87	(-10.02-8.29)	0.851					1.49	(-1.67-4.64)	0.354	2.09	(-0.19-4.37)	0.072	
Main problematic Substance: Alcohol	-20.10	(-34.47- -5.73)	0.006					-10.14	(-15.27–5.01)	< 0.001	-5.92	(-12.08-0.23)	0.058	
Main problematic Substance: Other	-10.17	(-19.50- -0.85)	0.033					2.30	(-0.99-5.60)	0.170	0.49	(-2.31-3.29)	0.728	
DASS 21 stress	-0.04	(-0.54-0.46)	0.875	-0.44	(-0.87- -0.01)	0.045		0.25	(0.06–0.44)	0.011				
DASS 21 depression	0.41	(-0.26-1.08)	0.217					0.25	(0.04–0.46)	0.020				
DASS 21 anxiety	-0.23	(-1.04-0.59)	0.583					0.11	(-0.21-0.44)	0.495				
Parental self-efficacy	-0.03	(-0.12-0.06)	0.462					-0.04	(-0.07–0.01)	0.011				

Reference variables for categorical predictors: Young adult level of education: In secondary school/vocational school; Employment young adult: Unemployed; Habitation young adult: With the participant; Parent relationship status: Married/Partnership/Cohabitant; and Main problematic substance: Cannabis.

* R2 square is pooled R-squares according to Snijders and Bosker (2012)

#### Relationship happiness

In the univariable analyses, the following predictors were found to correlate with an increase in relationship happiness rated by the parent over time (Table 5): baseline value of relationship happiness, where a higher level of relationship happiness predicted a higher level over time (*p* < 0.001), young adult being a student (*p* = 0.013) or working (*p* < 0.001) (compared to being unemployed or unknown employment status) and rating higher levels of parental self-efficacy at baseline (*p* < 0.001). The following predictors were correlated with lower relationship happiness over time: young adult previous treatment engagement (*p* = 0.049), and parental baseline levels of stress (*p* = 0.019).

In the multivariable model, only baseline value of relationship happiness was included, also here with higher baseline values predicting higher follow up scores, explaining 24% of the variance over time.

**Table 5:** Univariable and multivariable predictors of parental relationship happiness over 24 weeks post inclusion

Term	Univariable			Multivariable			
	Coef	95%-CI	*P*-value	Coef	95%-CI	*P*-value	R2*
							0.24
Relationship Happiness Scale	0.54	(0.41–0.67)	< 0.001	0.54	(0.41–0.67)	< 0.001	
Age young adult	-0.96	(-2.17-0.25)	0.120				
Age parent	0.32	(-0.08-0.71)	0.115				
*Young adult level of education* Primary school/dropped out of primary school	-1.42	(-11.28-8.43)	0.773				
Finished secondary school	-0.15	(-6.46-6.16)	0.964				
Dropped out of secondary school	-4.94	(-11.02-1.14)	0.111				
*Employment young adult* Studies	7.13	(1.50-12.76)	0.013				
Work	9.58	(4.17–14.98)	< 0.001				
*Habitation young adult* Every other week with parent/alone/with other	-1.78	(-6.88-3.32)	0.493				
Contact frequency parent-young adult	0.01	(-0.22-0.25)	0.900				
Young adult in treatment 180 − 30 days before baseline: yes	-5.91	(-11.80–-0.02)	0.049				
Parent relationship status: Single/divorced/widow/widower	2.30	(-2.79-7.39)	0.374				
Delivery mode: Face-to-face	1.22	(-3.69-6.12)	0.625				
Condition: CRAFT	-0.66	(-5.47-4.15)	0.787				
Main problematic Substance: Alcohol	-2.05	(-10.64-6.54)	0.638				
Main problematic Substance: Other	-2.24	(-8.01-3.52)	0.441				
DASS 21 stress	-0.34	(-0.63–-0.06)	0.019				
DASS 21 depression	-0.33	(-0.66-0.00)	0.053				
DASS 21 anxiety	-0.17	(-0.63-0.29)	0.475				
Parental self-efficacy	0.10	(0.05–0.15)	< 0.001				

Reference variables for categorical predictors: Young adult level of education: In secondary school/vocational school; Employment young adult: Unemployed; Habitation young adult: With the participant; Parent relationship status: Married/Partnership/Cohabitant; and Main problematic substance: Cannabis

* R2 square is pooled R-squares according to Snijders and Bosker (2012)

## Discussion

This present study is, to our knowledge, the first investigation of predictors of positive outcomes in a CRAFT-trial aimed at parents of substance using young adults. Main results from the analyses include that previous young adult treatment experience and higher parental self-efficacy were the strongest predictors for young adult treatment entry, while baseline level of young adult alcohol- or other substance use, and severity were the main predictor of alcohol-/substance use and severity over time. Lastly, baseline scores for relationship happiness were the strongest predictor of higher relationship happiness over time.

### Treatment entry

Regarding the primary outcome, the analysis of young adult treatment entry highlights the significant role of previous treatment engagement 180 − 30 days before baseline and parental self-efficacy as predictors. In the multivariable model, previous treatment engagement remained significant and was joined by higher parental self-efficacy as part of the best-fitting model, collectively explaining 16% of the variance.

Several different factors associated with a higher rate of treatment re-entry have been presented in previous studies, for example more severe substance use problems [40, 50]. Other studies suggest that positive prior experiences with treatment and familiarity with treatment processes can reduce psychological barriers to seek treatment, such as stigma or fear, making individuals more likely to seek help again [51]. Other findings have emphasized environmental factors such as more family/parental involvement and support as factors leading to increased treatment seeking [41, 50]. In the current trial, there were no significant associations between severity of substance use and treatment seeking, but instead, higher parental-self efficacy emerged as predictive of treatment entry. This result underscores the importance of confident and supportive parenting in influencing young adult behavior. Higher parental self-efficacy may translate into more effective communication, encouragement, and boundary-setting, which could motivate treatment engagement. These findings align with prior research highlighting the influence of parental self-efficacy on adolescent and young adult outcomes, including substance use behaviors and treatment adherence [52].

To our knowledge, no prior study on CSO support programs have investigated previous treatment engagement as a predictive factor of subsequent treatment entry. In most trials on CRAFT, reports on earlier treatment participation have been restricted to the IP not accepting treatment in the past 3–6 months since such engagement is argued to indicate that the IP is not treatment refusing (e.g [21, 22, 24, 27]), A Danish CRAFT trial identified only one significant predictor of treatment entry which was if the participating CSO had informed their IP that they participated in a CRAFT-program (adjusted OR = 2.29, CI: 1.13; 4.63, (*p* < 0.05) [35]. Whether the parents had informed the young adult of CRAFT participation was however not assessed in the current trial. A German study on CRAFT for CSOs of adults with alcohol use disorder found that CSOs primarily motivated by getting their relative into treatment were more successful in achieving this goal than those focused on their own well-being [24]. In the current study [36], one inclusion criterion was a strong desire for the young adult to enter treatment, which is why we did not include any measures of other potential motivational factors for participation in the study.

Although the final model explained a modest 16% of the variance, this reflects the complexity of treatment-seeking behavior, which is influenced by a multitude of individual, familial, and contextual factors. However, from a clinical perspective, enhancing parental self-efficacy emerges as a potential intervention target.

### Young adult alcohol use

The findings for young adult alcohol use showed similarities between the measures applied in the study (TLFB vs. AUDIT-C), but also differences in explanatory power. A key similarity was the consistent role of baseline alcohol consumption as the strongest predictor in both models, with higher initial alcohol use predicting higher continued use over time, aligning with previous trials on treatment for alcohol use disorders [53, 54].

However, differences emerged in the explanatory power of the models. The multivariable model for AUDIT-C explained a significantly larger proportion of variance (54%) compared to the TLFB model (18%). This discrepancy may reflect differences in the measurement constructs: AUDIT-C captures broader patterns of alcohol use, including frequency, quantity, and binge drinking [43], whereas TLFB in the current study focused solely on the number of days alcohol was consumed. The higher R² for AUDIT-C may indicate that it provided a more comprehensive assessment of drinking behaviors, capturing variance not accounted for by TLFB.

Young adult age was a significant predictor in both models, although the direction of association differed. In the TLFB multivariable analysis, older age predicted fewer days with alcohol use, whereas in the AUDIT-C univariable model, older age was associated with higher alcohol consumption. This divergence may reflect differences in how each outcome captures alcohol use behaviors, with TLFB measuring days with alcohol use the past month and not the volume consumed, while AUDIT-C captures a broader construct, including quantity on each occasion. In the RCT [36], parents receiving both CRAFT and manualized counselling reported significant reductions in days with alcohol use (TLFB) over time, while only a small decrease in AUDIT-C scores was reported in the counselling condition. It is hence possible that the divergence in variance explained could stem from the young adults consuming alcohol at fewer occasions over time, but still consuming large volumes on each occasion. This inference would align with research on patterns of binge drinking during the age from 18 to 24 across different countries [3, 4].

Univariable results for AUDIT-C identified a higher number of contextual predictors, including living arrangements, educational attainment, and the mode of parental support delivery. These were not significant in the TLFB analysis. This could suggest that AUDIT-C is more sensitive to variations in the social and environmental contexts influencing alcohol behaviors. This suggestion is supported by findings from a study on the predictive capacity of AUDIT-C among Finnish adolescents which demonstrated that AUDIT-C is effective in capturing contextual influences on adolescent alcohol use [55].

Overall, these findings underscore the value of using complementary approaches to assess alcohol-related outcomes, as they may capture distinct dimensions of drinking behavior and provide a fuller understanding of young adult alcohol use.

### Young adult substance use

The analyses of young adult substance use (measured by TLFB) and severity of substance use (measured by DUDIT) reveal both overlaps and distinctions in predictions between the two measures. As with alcohol use, a consistent finding across both outcomes is the predictive strength of baseline substance use and severity. In both models, higher initial levels of substance use and severity were the strongest predictors of continued use and higher severity over time. The predictive value of initial use and severity is consistent with previous research demonstrating the persistent nature of substance use behaviors, with baseline consumption and severity of substance use being the strongest predictor of use and severity in a three year follow-up of cannabis users [56] as well as for relapse amongst users of opioids and other substances [57]. The significant explanatory power of baseline use and severity underscores the importance of early detection and intervention to disrupt these patterns.

Another similarity is the modest contribution of additional predictors. For both TLFB and DUDIT, factors such as young adult age, parental stress, or the type of problematic substance showed univariable associations but were less impactful in the multivariable models. This suggests that while contextual factors may influence substance use behaviors, baseline use and severity remains the dominant explanatory factors.

Parental stress emerged as a noteworthy predictor for substance use and severity over time, with contrasting effects across models. Higher baseline parental stress predicted lower substance use as measured by TLFB, while for DUDIT as the outcome, higher parental stress correlated with increased severity of young adult substance use in univariable analyses.

These findings suggest that parental stress might prompt more active monitoring or intervention strategies, known to reduce adolescent substance use (e.g [58]), aligning with the TLFB analyses where stress was linked to decreased use. Conversely, the DUDIT analyses showed higher parental stress correlated with increased substance use severity over time. Elevated stress has been associated with negative, inconsistent, and coercive parenting behaviors (e.g [59], which predict higher young adult substance use and antisocial behaviors [60], possibly reflected in the DUDIT scores. These divergent findings highlight the complex interplay between parental stress and young adult substance use, necessitating further exploration.

Regarding DUDIT, older young adult age and higher parental self-efficacy emerged as protective factors in the univariable analysis. These findings are consistent with studies linking parental confidence in their ability to manage problematic behaviors to improved substance use outcomes [52]. While the final model underscores the importance of baseline severity of young adult substance use, the role of parental and psychosocial factors in shaping substance use trajectories remains an important avenue for further research.

### Relationship happiness

The analysis of parental relationship happiness over time highlights the central role of baseline relationship happiness, with higher initial scores predicting higher follow-up levels (*p* < 0.001), explaining 24% of the variance in the multivariable model.

Univariable analyses revealed additional predictors associated with changes in relationship happiness. For instance, higher young adult engagement in work or studies was linked to increased parental relationship happiness, suggesting that parents perceive their young adult’s productive activities as positive for family well-being. This supports findings in prior literature that parental satisfaction often increases when adult children demonstrate independence or goal-oriented behaviors (e.g [61]). Additionally, higher baseline parental self-efficacy was positively correlated with relationship happiness, reinforcing evidence that confident parenting improves family dynamics [62].

Conversely, stress and previous treatment engagement by the young adult were associated with lower relationship happiness in univariable analyses. The link between stress and diminished relationship satisfaction is well-documented, with parental stress often increasing family tensions (e.g [63]). Similarly, previous treatment engagement may reflect more complex or ongoing challenges in the young adult’s life, potentially contributing to strain in the parent-child relationship [64, 65] and, consequently, lower relationship happiness.

Despite these findings, only baseline relationship happiness remained significant in the multivariable model. This suggests that while other factors may influence relationship happiness, initial levels are the strongest predictor of trajectories over time. This aligns with prior studies highlighting that baseline relationship satisfaction often moderates responses to interventions or life changes [61]. Nevertheless, the results provide arguments for addressing parental stress and promoting parental self-efficacy and young adult independence through engagement in work or studies as potential pathways to enhance relationship satisfaction.

### Strengths and limitations

The mixed-effects regression models used in this study offer several advantages, including accounting for intra-individual variability over time and enabling the analysis of within- and between-subject effects while accommodating missing data. The application of multiple imputation (MICE) reduced bias from missing data, and the stepwise selection process, guided by the Akaike Information Criterion (AIC), ensured model parsimony and retention of the best predictors.

The methodological approach has limitations. The exploratory nature of the analyses increases the risk of overfitting due to the number of predictors relative to the sample size. The outcome data regarding days with alcohol/substance use (TLFB), were highly skewed. To address this, we applied log-transformation to improve model fit and interpretability. Nonetheless, relying on mean-based outcomes may have obscured clinically meaningful effects in potential subgroups. However, the modest sample size limited our ability to conduct reliable subgroup analyses, but we recognize that future studies should consider incorporating clinically meaningful change thresholds where available. The limitation in sample size is particularly notable for alcohol-related measures, as few participants identified alcohol as their young adult’s primary problem, limiting statistical power.

Although TLFB typically includes both frequency and quantity, we chose to capture only days of use to ensure feasibility and consistency across substances. Estimating drug quantities per occasion was considered too challenging for the parents, potentially compromising data quality. While this decision may have reduced sensitivity to detect changes in alcohol use—since consumption can vary widely between days—we applied the same approach for all substances, for the sake of comparability across substance-related outcomes.

A potential limitation concerns the differing time frames across outcome measures. TLFB assessed substance use over the past month, whereas AUDIT-C and DUDIT captured patterns and related problems over the past year—thus overlapping with pre-intervention behavior. This broader recall period may reduce sensitivity to change, as it blends pre- and post-intervention patterns. As such, AUDIT-C and DUDIT may be less responsive to short-term effects compared to TLFB. Future studies should aim to align time frames across measures to enhance interpretability and validity.

Another potential limitation is that increased parental awareness, fostered by the CRAFT program’s focus on monitoring substance use, may have led to higher reporting without reflecting actual behavioral change. Hence, parents in the CRAFT condition might have become more attentive to signs of use over time, potentially inflating reported substance use even if the young adult’s behavior remained stable.

The gender imbalance among participants, with mothers vastly overrepresented, reflects patterns seen in previous CSO studies (e.g [34], but limits our ability to examine potential differences in outcomes based on parent gender. Although fathers were often involved in sessions in our study, they were not the registered participants, and their involvement was not systematically documented. Future research should explore strategies to engage fathers more effectively to support youth and promote balanced family dynamics.

Although parental self-efficacy emerged as a significant predictor of young adult treatment entry, the degree to which this construct is modifiable through interventions remains uncertain. The self-efficacy measure used in the current study includes multiple domains which may may differ in their malleability as outcomes in an intervention. However, even modest improvements in perceived parenting competence may enhance parent–child communication and boundary-setting, potentially supporting positive behavioral change in the young adult. Future research is needed to disentangle which aspects of self-efficacy are most amenable to change and how these relate to family dynamics and clinical outcomes in populations affected by substance use.

All predictors were assessed through parent-report only, which may introduce bias, particularly for variables dependent on the level of contact and interaction. For example, substance use in young adults not living at home may be more difficult for parents to accurately observe. This reliance on subjective reports limits the possibility to corroborate with objective measures. However, prior research supports the validity of parental reports in this context, indicating acceptable correspondence with adolescent self-reports of substance use [66, 67].

Inferences regarding prior treatment engagement are limited by the six-month assessment window before study initiation, excluding earlier treatment experiences.

Finally, the high level of education among the participants, and low numbers of participants born in other countries, were not representative of the Swedish general population and could potentially decrease the generalizability of the findings.

## Conclusions

This exploratory study found that prior treatment engagement and higher parental self-efficacy predicted treatment initiation, highlighting parental self-efficacy as a key intervention target. Baseline use and severity consistently predicted higher young adult alcohol- and substance use and severity over time, emphasizing the importance of early detection and intervention. Baseline relationship happiness was the strongest predictor of future relational happiness. Future research should focus on enhancing parental self-efficacy and addressing parental stress to improve family dynamics and substance use outcomes.

## Data Availability

No datasets were generated or analysed during the current study.

## Abbreviations

AFT: Al-anon facilitation therapy
AUD: Alcohol use disorder
AUDIT-C: Alcohol use disorders identification test-consumption
CBT: Cognitive behavioral therapy
CRAFT: Community reinforcement and family training
CSO: Concerned significant other
CST: Coping skills training
DASS-21: Depression, anxiety, and stress scale
DUDIT: Drug use disorder identification test
IP: Identified patient
MICE: Multiple imputation by chained equations
MI: Motivational interviewing
OR: Odds ratio
RCT: Randomized controlled trial
RHS: Relationship happiness scale
SUD: Substance use disorder
TLFB: TimeLine followback
